# Estimating mono- and bi-phasic regression parameters using a mixture piecewise linear Bayesian hierarchical model

**DOI:** 10.1371/journal.pone.0180756

**Published:** 2017-07-19

**Authors:** Rui Zhao, Paul Catalano, Victor G. DeGruttola, Franziska Michor

**Affiliations:** 1 Department of Biostatistics, Harvard School of Public Health, Boston, Massachusetts 02115, United States of America; 2 Department of Biostatistics and Computational Biology, Dana-Farber Cancer Institute, Boston, Massachusetts 02215, United States of America; Indiana University Bloomington, UNITED STATES

## Abstract

The dynamics of tumor burden, secreted proteins or other biomarkers over time, is often used to evaluate the effectiveness of therapy and to predict outcomes for patients. Many methods have been proposed to investigate longitudinal trends to better characterize patients and to understand disease progression. However, most approaches assume a homogeneous patient population and a uniform response trajectory over time and across patients. Here, we present a mixture piecewise linear Bayesian hierarchical model, which takes into account both population heterogeneity and nonlinear relationships between biomarkers and time. Simulation results show that our method was able to classify subjects according to their patterns of treatment response with greater than 80% accuracy in the three scenarios tested. We then applied our model to a large randomized controlled phase III clinical trial of multiple myeloma patients. Analysis results suggest that the longitudinal tumor burden trajectories in multiple myeloma patients are heterogeneous and nonlinear, even among patients assigned to the same treatment cohort. In addition, between cohorts, there are distinct differences in terms of the regression parameters and the distributions among categories in the mixture. Those results imply that longitudinal data from clinical trials may harbor unobserved subgroups and nonlinear relationships; accounting for both may be important for analyzing longitudinal data.

## Introduction

Mixed-effects models are particularly useful in medical research because of their ability to handle imbalances in the number of observations across patients and to identify between-subject and within-subject sources of variability [1–3]. Progress to extend mixed-effects models to include heterogeneity in data has been made by incorporating a finite mixture into the model [4, 5]. This extension is particularly relevant to clinical research, since clinical data often contain unobserved categorical variables corresponding to, for example, “responders” or “non-responders” to a given treatment. Ignoring such mixtures may result in biases in estimates. Xu and Hedeker investigated this idea and found there is ample evidence of non-homogeneous responses in two large psychiatric clinical trials [6]. Ketchum *et al.* further extended the mixed-effects mixture models to allow for differences in the variance-covariance matrices [7]. These improvements enable the random-effects models to better characterize heterogeneity in data. A book chapter by Verbeke and Molenberghs provides an excellent summary of heterogeneous mixed models [8].

In addition to population heterogeneity, changes in functional relationships between response variables and explanatory variables, particularly with time, are ubiquitous in longitudinal studies: HIV-1 viral load [9, 10], hepatitis B/C viral load [11, 12] and BCR-ABL expression levels in chronic myeloid leukemia [13, 14]. In those examples, biomarkers exhibit nonlinear changes over time, many of which are bi-phasic in nature—that is, patients biomarkers have two distinct patterns over time rather than one uniform trajectory. An example of bi-phasic decline patterns is that in some chronic myeloid leukemia patients, the initial decline of BCR-ABL expression levels is much faster than later declines, whereas in other patients the decline is uniform over time [13, 14]. These observations are contrary to the assumptions of many models that parameters are invariant over time. One method for accounting for changes in the longitudinal relationships over time is provided by nonlinear mixed-effects models [15, 16]. Morrel *et al.* applied a piecewise nonlinear mixed-effects model to a prostate cancer data set and found that patients with local lesions and metastatic lesions have similar initial prostate-specific antigen (PSA) trajectories. However, they found that the rates of PSA increase in a later phase were larger in patients with metastatic lesions than patients with local lesions. Naumova *et al.* used a piecewise mixed-effect model to analyze a prospective study on the development of obesity in female adolescents [17]. Cudeck and Klebe, and Harring *et al.* applied similar ideas to psychology-related data sets [18, 19]. Those examples demonstrate the flexibility of nonlinear mixed-effects models in investigating changing functional relationships over time.

Both heterogeneity in patient populations and changes in the longitudinal relationship have been addressed separately in several publications [6, 7, 15–19]; however, only a few publications have tackled both problems simultaneously. Pauler and Laird introduced a general framework for finite mixtures of nonlinear hierarchical models; they applied their methods to investigate non-compliance in a HIV clinical trial [20]. In their application, the mixture consists of a constant mean model for the compliant patients and a piecewise linear model for the non-compliant patients. Recently, Lu and Huang extended the general framework proposed by Pauler and Laird [20] to incorporate skewness in the distributions of individual regression parameters, relaxing the normal assumption [21]; they applied their methods to analyze a HIV viral load data set [22]. Their underlying nonlinear mixed-effects model was formulated based on the model structure of an ordinary differential equation (ODE) model describing viral loads over time [23]. One caveat associated with those approaches is that their models require extensive prior biological knowledge in order to specify the nonlinear models before analyzing the data. Misspecification of the model may have detrimental effects on parameter estimation and patient classification. Particularly, if the differences between categories in the mixture are not well separated, specifying the model becomes an even more challenging problem.

To address this issue, we developed a piecewise linear random-effects mixture model that does not require any prior knowledge on the model structure to account for both heterogeneity and change in the longitudinal relationship over time. The only assumptions of this model are that the underlying data may contain a mixture of mono- and bi-phasic observations, and that the bi-phasic observations are piecewise linear; no further constraints on the intercepts and slopes are necessary. The primary purpose of this model is to detect unobserved subgroups in a patient population and nonlinear longitudinal relationships over time. This method is particularly useful for current clinical trials, which often include diagnostic and prognostic hypotheses. With periodical follow-up visits gathering biomarker data, parameters associated with heterogeneous changes in biomarker trends can be detected with this method. Given the usually limited number of follow-up measurements in clinical trials, the current implementation of this model focuses primarily on mono- vs. bi-phasic changes; however, our model can easily be generalized to include multi-phasic changes and multi-category mixtures. In addition, because of the piecewise linear nature of the proposed model, other clinically relevant covariates can also easily be included.

## Materials and methods

We designed a mixture piecewise linear Bayesian hierarchical model to estimate regression parameters and to determine the posterior distributions of these parameters, while accounting for both population heterogeneity and changes in longitudinal relationships over time. We consider situations in which the patient population consists two latent subpopulations: mono-phasic and bi-phasic patients. The individual-level trajectories for mono-phasic and bi-phasic patients are shown in Eq (1). Throughout the text, we use subscripts S and B to denote mono- (i.e. single-) and bi-phasic patients, respectively. For the *i*^*th*^ patient with a total of *M*_*i*_ observations, the dependent variable *y*_*ij*_, corresponding to the quantitative measure of disease burden, which may either follow a mono-phasic or a bi-phasic regression line, depending on the latent indicator variable *η*_*i*_:

$$\begin{array}{cc} & \eta_{i}=0:y_{ij}=s_{0i}+s_{1i}t_{ij}+\varepsilon_{ij}\text{,}\;\text{for}\;j=1...M_{i}\\ & \eta_{i}=1:\{\begin{array}{c} y_{ij}=b_{0i}+b_{1i}t_{ij}+\varepsilon_{ij}\text{,}\;\text{for}\;j=1...k_{i}\\ y_{ij}=b_{0i}^{\prime }+b_{1i}^{\prime }t_{ij}+\varepsilon_{ij}\text{,}\;\text{for}\;j=k_{i+1}...M_{i}. \end{array} \end{array}$$ (1)

Here, *ε*_*ij*_ denotes the independent error term, which follows a normal distribution centered at 0 with variance *σ*^2^; *η*_*i*_ denotes the phasic indicator for patient *i*, with 0 and 1 denoting mono- and bi-phasic patterns, respectively; *k*_*i*_, a latent variable, denotes the number of observations belonging to the first phase for patient *i*, if the response of patient *i* is bi-phasic. For the individual regression parameters, we assume hierarchical normal distributions for *s*_*i*_ and *b*_*i*_:

$$\begin{array}{c} s_{i}=\left(\begin{array}{c} s_{0i}\\ s_{1i} \end{array}\right)\overset{iid}{∼}N\left(\begin{array}{c} \left(\begin{array}{c} S_{0}\\ S_{1} \end{array}\right),\Sigma_{S,2\times 2} \end{array}\right)=N\left(S,\Sigma_{S}\right) \end{array}$$ (2)

$$\begin{array}{c} b_{i}=\left(\begin{array}{c} b_{0i}\\ b_{1i}\\ b_{0i}^{\prime }\\ b_{1i}^{\prime } \end{array}\right)\overset{iid}{∼}N\left(\begin{array}{c} \left(\begin{array}{c} B_{0}\\ B_{1}\\ B_{0}^{\prime }\\ B_{1}^{\prime } \end{array}\right),\Sigma_{B,4\times 4} \end{array}\right)=N\left(B,\Sigma_{B}\right) \end{array}$$ (3)

We first consider the artificial case in which we do not know if a patient follows the mono- or bi-phasic pattern, but if this patient follows a bi-phasic pattern, the associated bi-phasic design matrix is known. That is, for each patient regardless phasicity, the true mono- and bi-phasic design matrices are known; the only unknown quantity is the phasicity, *η*_*i*_. Assuming the prior distributions *P*(*σ*^2^) = (*σ*^2^)^−1^, *P*(*λ*) = *Beta*(1, 1) = 1, *P*(Σ_*S*_) = |Σ_*S*_|^−(2+1)/2^ and *P*(Σ_*B*_) = |Σ_*B*_|^−(4+1)/2^, where *λ* denotes the proportion of bi-phasic patients and *η*_*i*_ ∼ *Ber*(*λ*), the posterior distribution is:

$$\begin{array}{ccc} & & P\left(S,\Sigma_{S},B,\Sigma_{B},\sigma^{2},\lambda |Y,\eta \right)\propto {\left(\sigma^{2}\right)}^{-1}\Sigma_{S}^{-(2+1)/2}\Sigma_{B}^{-(4+1)/2}\\ & & \prod_{i=1}^{N}{\left\{\left(1-\lambda \right)P\left(Y_{i}|Q_{i}^{s}S,Q_{i}^{s}\Sigma_{S}{\left(Q_{i}^{s}\right)}^{T}+I\sigma^{2}\right)\right\}}^{1-\eta_{i}}\prod_{i=1}^{N}{\left\{\lambda P\left(Y_{i}|Q_{i}^{b}B,Q_{i}^{b}\Sigma_{B}{\left(Q_{i}^{b}\right)}^{T}+I\sigma^{2}\right)\right\}}^{\eta_{i}}. \end{array}$$ (4)

Here *η* = (*η*_1_, …, *η*_*N*_) is the missing indicator variable for phasicity, and $Q_{i}^{s}$ and $Q_{i}^{b}$ denote the individual mono- and bi-phasic design matrices, respectively:

$$\begin{array}{c} Q_{i}^{s}=\left[\begin{array}{cc} 1 & t_{i1}\\ \vdots & \vdots \\ 1 & t_{ik}\\ 1 & t_{ik+1}\\ \vdots & \vdots \\ 1 & t_{iM_{i}} \end{array}\right] \end{array}$$ (5)

$$\begin{array}{c} Q_{i}^{b}=\left[\begin{array}{cccc} 1 & t_{i1} & 0 & 0\\ \vdots & \vdots & \vdots & \vdots \\ 1 & t_{ik} & 0 & 0\\ 0 & 0 & 1 & t_{ik+1}\\ \vdots & \vdots & \vdots & \vdots \\ 0 & 0 & 1 & t_{iM_{i}} \end{array}\right] \end{array}$$ (6)

However, the bi-phasic design matrix for subject *i*, $Q_{i}^{b}$, is not known. The estimation of this bi-phasic change point is a well-known problem in statistics, mathematics, and computer science with many applications in other fields; several methods for addressing this question have been suggested [24, 25]. Here we employed a Bayesian formulation of the change point problem, suggested by Carlin *et al.* [26]. For a particular patient *i* with *M*_*i*_ observations, there are *M*_*i*_ − 1 possible change points. The cases in which the bi-phasic transition point occurs before the first observation or after the last observation are ignored, because in such cases mono- and bi-phasic subjects are not distinguishable. Thus, for each patient *i*, there are *M*_*i*_ − 1 possible design matrices, for example:

$$\begin{array}{c} Q_{i1}^{b}=\left[\begin{array}{cccc} 1 & t_{i1} & 0 & 0\\ 0 & 0 & 1 & t_{i2}\\ 0 & 0 & 1 & t_{i3}\\ \vdots & \vdots & \vdots & \vdots \\ 0 & 0 & 1 & t_{iM_{i}} \end{array}\right] \end{array}$$ (7)

$$\begin{array}{c} Q_{i2}^{b}=\left[\begin{array}{cccc} 1 & t_{i1} & 0 & 0\\ 1 & t_{i2} & 0 & 0\\ 0 & 0 & 1 & t_{i3}\\ \vdots & \vdots & \vdots & \vdots \\ 0 & 0 & 1 & t_{iM_{i}} \end{array}\right] \end{array}$$ (8)

For each corresponding design matrix $Q_{ij}^{b}$, the probability associated with the *j*-th bi-phasic design matrix is denoted by *π*_*ij*_. Assuming all patients comply with their clinic visit schedules, such that *t*_11_ = *t*_21_ = … = *t*_*N*1_, …, and *t*_1*M*_1__ = *t*_2*M*_2__ = … = *t*_*NM*_*N*__, and assuming an uninformative Dirichlet prior, Dir(*α*_1_ = *α*_2_ = … = *α*_*M*_*i*_−1_ = 1), the posterior function is:

$$\begin{array}{ccc} & & P\left(S,B,\Sigma_{S},\Sigma_{B},\lambda ,\pi ,\sigma^{2}|Y,\xi ,\eta \right)\propto {\left(\sigma^{2}\right)}^{-1}\Sigma_{S}^{-(2+1)/2}\Sigma_{B}^{-(4+1)/2}\\ & & \prod_{i=1}^{N}[\left(1-\lambda \right)N(Y_{i}|Q_{i}^{s}S,Q_{i}^{s}\\ & & {\Sigma_{S}{\left(Q_{i}^{s}\right)}^{T}+I\sigma^{2})]}^{1-\eta_{i}}{\left[\lambda \prod_{j=1}^{M_{i}-1}{\left\{\pi_{ij}N\left(Y_{i}|Q_{ij}^{b}B,Q_{ij}^{b}\Sigma_{B}{\left(Q_{ij}^{b}\right)}^{T}+I\sigma^{2}\right)\right\}}^{\xi_{ij}}\right]}^{\eta_{i}} \end{array}$$ (9)

where *ξ*_*ij*_ is the unobserved indicator for the *j*^*th*^ bi-phasic design matrix for subject *i*, such that $\sum_{j=1}^{M_{i}-1}\xi_{ij}=1$ and *ξ*_*ij*_ ∼ *Multinomial*(*π*_*ij*_), and *ξ*_*i*_ = (*ξ*_*i*0_, …, *ξ*_*iM*_*i*__) and *ξ* = (*ξ*_1_, …, *ξ*_*N*_); and *π*_*ij*_ is the probability that the *j*-th bi-phasic design matrix is selected for the i-th patient. Note that the inclusion of the Dirichlet prior results in a constant scaling factor, and hence it is not included in Eq (9).

Given the complexity of the model, we first used the Expectation Maximization (EM) algorithm to search for the mode of the posterior distribution, which was then used as the starting value for the Gibbs sampler. To implement the EM algorithm and to obtain Empirical Bayes estimators, we utilized the procedures derived by Verbeke and Lesaffre [5] and Xu and Hedeker [6]. Following the notation used in Xu and Hedeker, the Empirical Bayes estimators for individual regression parameters and the covariance matrices are given by

$$\begin{array}{ccc} \hat{s_{i}} & = & S+{\left(\Sigma_{S}^{-1}+{\left(Q_{i}^{s}\right)}^{T}{\left(\sigma^{2}I_{i}\right)}^{-1}Q_{i}^{s}\right)}^{-1}Q_{i}^{s}{\left(\sigma^{2}I_{i}\right)}^{-1}\left(Y_{i}-Q_{i}^{s}S\right)\\ {\hat{\Sigma }}_{s_{i}} & = & {\left(\Sigma_{S}^{-1}+{\left(Q_{i}^{s}\right)}^{T}{\left(\sigma^{2}I_{i}\right)}^{-1}Q_{i}^{s}\right)}^{-1}\\ {\hat{b}}_{ij} & = & B+{\left(\Sigma_{B}^{-1}+{\left(Q_{ij}^{b}\right)}^{T}{\left(\sigma^{2}I_{i}\right)}^{-1}Q_{ij}^{b}\right)}^{-1}Q_{ij}^{b}{\left(\sigma^{2}I_{i}\right)}^{-1}\left(Y_{i}-Q_{ij}^{b}B\right)\\ {\hat{\Sigma }}_{b_{ij}} & = & {\left(\Sigma_{B}^{-1}+{\left(Q_{ij}^{b}\right)}^{T}{\left(\sigma^{2}I_{i}\right)}^{-1}Q_{ij}^{b}\right)}^{-1}. \end{array}$$ (10)

for a given set of *S*, *B*, Σ_*S*_, and Σ_*B*_.

In the expectation step, the quantity

$$\begin{array}{c} \zeta_{ij}=P\left(\xi_{ij}=1|B,\Sigma_{B},\sigma^{2},Y_{i}\right)=\frac{\pi_{ij}N\left(Y_{i}|Q_{ij}^{b}B,Q_{ij}^{b}\Sigma_{B}{\left(Q_{ij}^{b}\right)}^{T}+I\sigma^{2}\right)}{\sum_{j=1}^{M_{i}-1}\pi_{ij}N\left(Y_{i}|Q_{ij}^{b}B,Q_{ij}^{b}\Sigma_{B}{\left(Q_{ij}^{b}\right)}^{T}+I\sigma^{2}\right)} \end{array}$$ (11)

is calculated. *ζ*_*ij*_ denotes the probability of the *j*-th bi-phasic matrix for the *i*-th patient is selected to be bi-phasic design matrix, and *ζ*_*i*_ = (*ζ*_*i*1_, *ζ*_*i*2_…*ζ*_*iM*_*i*__). Similarly, the expected value for *η*_*i*_ can be calculated as

$$\begin{array}{c} z_{i}=P\left(\eta_{i}=1|S,B,\Sigma_{S},\Sigma_{B},\lambda ,\sigma^{2},Y_{i}\right)=\frac{\lambda exp(\text{Eq(}14\text{)})}{\left(1-\lambda )exp(\text{Eq(}13\text{)})+\lambda exp(\text{Eq(}14\text{)}\right)}, \end{array}$$ (12)

where

$$\begin{array}{c} 2logN(Y_{i}|Q_{i}^{s}S,Q_{i}^{s}\Sigma_{S}{\left(Q_{i}^{s}\right)}^{T}+I\sigma^{2}) \end{array}$$ (13)

$$\begin{array}{c} 2log(\sum_{j=1}^{M_{i}-1}\pi_{ij}N\left(Y_{i}|Q_{ij}^{b}B,Q_{ij}^{b}\Sigma_{B}{\left(Q_{ij}^{b}\right)}^{T}+I\sigma^{2}\right)) \end{array}$$ (14)

and where Eqs (13) and (14) are the posteriors for the mono- and bi-phase pieces. However, in practice, given the added model complexity of the bi-phasic model compared to the mono-phasic model, the bi-phasic model has a larger likelihood than the single phasic model, resulting in most patients being classified as bi-phasic. To compensate for the difference in model complexity, we instead of using Eqs (13) and (14) to differentiate each patient’s phasicity, we used the negative Bayesian Information Criteria (BIC) for the mono- and bi-phasic models, respectively:

$$\begin{array}{c} 2logN(Y_{i}|Q_{i}^{s}S,Q_{i}^{s}\Sigma_{S}{\left(Q_{i}^{s}\right)}^{T}+I\sigma^{2})-2log\left(M_{i}\right) \end{array}$$ (15)

$$\begin{array}{c} 2log\left(\sum_{j=1}^{M_{i}-1}\pi_{ij}N\left(Y_{i}|Q_{ij}^{b}B,Q_{ij}^{b}\Sigma_{B}{\left(Q_{ij}^{b}\right)}^{T}+I\sigma^{2}\right)\right)-4log\left(M_{i}\right), \end{array}$$ (16)

The additional terms, −2*log*(*M*_*i*_) and −4*log*(*M*_*i*_) are constant factors, which can be seen as prior odds for distinguishing between mono- and bi-phasic models for each patient. Because they are constants, they only result in a proportional change in the posterior function, Eq (9). Similar methods of using BIC to determine the posterior model probabilities have been implemented and discussed by Kass and Raftery [27]. BIC corrects for the improvement in fitting associated with increasing model complexity and BIC has been shown to be a consistent model selector due to its quickly increasing penalty as a function of the sample size [28–30]. We also investigated AIC as an alternative penalty function. However, as the sample size increases, the penalty becomes too weak, resulting in mono-phasic patients being misclassified as bi-phasic patients. In addition, the use of BIC for model selection is analogous to the use of DIC (Deviance Information Criterion) in Bayesian mixture model [31].

The maximization consists of the following steps:

$$\begin{array}{ccc} & & {\hat{\lambda }}^{new}=\frac{1}{N}\sum_{i=1}^{N}z_{i}\\ & & {\hat{S}}^{new}=\frac{\sum_{i=1}^{N}\left(1-z_{i}\right){\hat{s}}_{i}}{\sum_{i=1}^{N}\left(1-z_{i}\right)}\\ & & {\hat{\Sigma }}_{S}^{new}=\frac{\sum_{i=1}^{N}\left(1-z_{i}\right)\left({\hat{\Sigma }}_{s_{i}}+\left({\hat{s}}_{i}-{\hat{S}}^{new}\right){\left({\hat{s}}_{i}-{\hat{S}}^{new}\right)}^{T}\right)}{\sum_{i=1}^{N}\left(1-z_{i}\right)+2}\\ & & {\hat{B}}^{new}=\frac{\sum_{i=1}^{N}\sum_{j=0}^{M_{i}}z_{i}\zeta_{ij}{\hat{b}}_{ij}}{\sum_{i=1}^{N}z_{i}}\\ & & {\hat{\Sigma }}_{B}^{new}=\frac{\sum_{i=1}^{N}\sum_{j=0}^{M_{i}}z_{i}\zeta_{ij}\left({\hat{\Sigma }}_{b_{ij}}+\left({\hat{b}}_{ij}-{\hat{B}}^{new}\right){\left({\hat{b}}_{ij}-{\hat{B}}^{new}\right)}^{T}\right)}{\sum_{i=1}^{N}z_{i}+2}\\ & & {\hat{\sigma }}^{2new}=\frac{\sum_{i=1}^{N}\left(1-z_{i}\right)\left[u_{i}^{s}{\left(u_{i}^{s}\right)}^{T}+Q_{i}^{s}{\hat{\Sigma }}_{s_{i}}{\left(Q_{i}^{s}\right)}^{T}\right]}{\sum_{i=1}^{N}\left(1-z_{i}\right)+2}+\frac{\sum_{i=1}^{N}\sum_{j=0}^{M_{i}}z_{i}\zeta_{ij}\left[u_{ij}^{b}{\left(u_{ij}^{b}\right)}^{T}+Q_{ij}^{b}{\hat{\Sigma }}_{b_{ij}}{\left(Q_{ij}^{b}\right)}^{T}\right]}{\sum_{i=1}^{N}z_{i}+2}, \end{array}$$ (17)

where $u_{ij}^{b}=Y_{i}-Q_{ij}^{b}{\hat{b}}_{ij}$.

Updating the probability weight for *π*_*ij*_, if all patients adhere to the visit schedule, is straightforward by averaging *ζ*_*ij*_. However, in practice, patients often miss scheduled visits entirely or have unscheduled visits. Such departure from the trial design creates misalignments in patients’ observation intervals. For instance, two bi-phasic patients, *i* and *i*′, are identical except for their *j* + 1^*th*^ visits. Patient *i*’s *j* + 1^*th*^ visit is 1 week later than the scheduled time and patient *i*′ is on time. Because of this difference in visit time, *ζ*_*ij*_ and *ζ*_*i*′*j*_ can no longer be simply averaged to update *π*_*ij*_. Instead, to account for misalignments, the transition probability *π*_*ij*_ associated with bi-phasic transition design matrix *Q*_*ij*_ needs to adapt to each patient’s actual visit time. The phasic transition density as a function of time is

$$\begin{array}{c} \theta \left(t\right)=\frac{\sum_{i=1}^{N}\sum_{j=1}^{M_{i}-1}\zeta_{ij}I\left(t_{ij}<t<t_{i(j+1)}\right)\eta_{i}}{\int_{0}^{T}\sum_{i=1}^{N}\sum_{j=1}^{M_{i}-1}\zeta_{ij}I\left(t_{ij}<t<t_{i(j+1)}\right)\eta_{i}dt}\text{.} \end{array}$$ (18),

where *T* denotes the maximum follow-up time for all patients. The denominator is the weighted sum of time interval lengths in which phasic transitions occur; this denominator serves as the normalizing factor to ensure *θ*(*t*) integrates to 1. Eq (14) results in a numeric stepwise function specifying the transition density at a given t based on the E-step. The weight for each interval for each patient can be updated by integrating over its corresponding interval:

$$\begin{array}{c} \pi_{ij}=\int_{t_{ij}}^{t_{i(j+1)}}\theta \left(t\right)dt\text{.} \end{array}$$ (19)

The starting values for the EM algorithm are obtained using an *ad hoc* grid search procedure as outlined in S1 File.

The estimated parameter mode from the EM algorithm are used as the starting values for the Gibbs’ sampler to simulate the posterior distributions of the parameters from the model specified in Eq (9), as outlined below:

1. Calculate *ζ*_*i*_ for each patient, using Eq (11).
2. For each patient, draw a *ξ*_*i*_ vector from a multinomial distribution with a parameter vector *ζ*_*i*_, and obtain the corresponding bi-phasic design matrix $Q_{ij}^{b}$, such that *ξ*_*ij*_ = 1.
3. Calculate *z*_*i*_ based on the mono-phasic design matrix $Q_{i}^{s}$ and the bi-phasic design matrix $Q_{ij}^{b}$ from step (2).
4. Draw *η*_*i*_ from a Bernoulli distribution with parameter *z*_*i*_ for each patient.
5. Update *θ*(*t*) using Eq (18), with *ζ*_*ij*_ replaced by *ξ*_*ij*_ and *z*_*i*_ replaced by *η*_*i*_.
6. Draw a vector *π*_*i*_ from a Dirichlet distribution with a parameter vector $\left(\int_{t_{i1}}^{t_{i2}}\theta \left(t\right)dt+1,...,\int_{t_{iM_{i}-2}}^{t_{iM_{i}-1}}\theta \left(t\right)dt+1\right)$, for each patient.
7. Draw *λ* from a Beta distribution with parameters ($\sum_{i=1}^{N}\eta_{i}+1$, $\sum_{i=1}^{N}\left(1-\eta_{i}\right)+1$).
8. Sampling *s*_*i*_ and *b*_*i*_:
  $$\begin{array}{ccc} s_{i} & ∼ & N({\hat{s}}_{i},{\hat{\Sigma }}_{s_{i}})\\ b_{i} & ∼ & N({\hat{b}}_{ij},{\hat{\Sigma }}_{b_{ij}})) \end{array}$$ (20)
  where ${\hat{s}}_{i}$ and ${\hat{\Sigma }}_{s_{i}}$ and ${\hat{b}}_{ij}$ and ${\hat{\Sigma }}_{b_{ij}}$ are from Eq (12). The design matrix for the bi-phasic is drawn from step (2).
9. Sampling *S* and *B* from *s*_*i*_ and *b*_*i*_:
  $$\begin{array}{ccc} S & ∼ & N(\frac{\sum_{i=1}^{N}\left(1-\eta_{i}\right)s_{i}}{\sum_{i=1}^{N}\left(1-\eta_{i}\right)},\frac{\Sigma_{S}}{\sum_{i=1}^{N}\left(1-\eta_{i}\right)})\\ B & ∼ & N(\frac{\sum_{i=1}^{N}\eta_{i}b_{i}}{\sum_{i=1}^{N}\eta_{i}},\frac{\Sigma_{B}}{\sum_{i=1}^{N}\eta_{i}}) \end{array}$$ (21)
10. Sampling Σ_*S*_ and Σ_*B*_:
  $$\begin{array}{ccc} \Sigma_{S} & ∼ & Inv-Wishart(\sum_{i=1}^{N}\left(1-\eta_{i}\right)-1,\sum_{i=1}^{N}\left(1-\eta_{i}\right)\left(s_{i}-S\right){\left(s_{i}-S\right)}^{t})\\ \Sigma_{B} & ∼ & Inv-Wishart(\sum_{i=1}^{N}\left(\eta_{i}\right)-3,\sum_{i=1}^{N}\left(\eta_{i}\right)\left(b_{i}-B\right){\left(b_{i}-B\right)}^{t}) \end{array}$$ (22)
11. Sampling *σ*^2^:
  $$\begin{array}{ccc} & & Inv-\chi^{2}(\sum_{i=1}^{N}M_{i},\\ & & \;\frac{1}{\sum_{i=1}^{N}M_{i}}\sum_{i=1}^{N}\left\{\left(1-\eta_{i}\right){\left(Y_{i}-Q_{i}^{s}s_{i}\right)}^{t}\left(Y_{i}-Q_{i}^{s}s_{i}\right)+\left(\eta_{i}\right){\left(Y_{i}-Q_{i}^{b}b_{i}\right)}^{t}\left(Y_{i}-Q_{i}^{b}b_{i}\right)\right\}) \end{array}$$ (23)

The proposed Gibbs’ sampler is similar to the methods used for variable selection via Gibbs sampling proposed by George and McCulloch [32], and, Carlin and Chib [33]. The only difference between our methods and the model by Carlin and Chib is the lack of pseudoprior for the transition probability between mono- and bi-phasic models. An important consequence of this is that, as noted in Carlin and Chib’s publication, “it is tempting to skip the generation of actual pseudoprior values … although seemingly reasonable, such an algorithm is clearly not a Gibbs sampler in the strict sense, since the nodes visited are determined by the current value in the realized Markov Chain.” However, in practice, as shown by our simulation studies, this heuristic Gibbs sampler performs well. Other methods such as reversible-jump MCMC may also be used to sample the posterior distributions [34]; however given the simplicity of the Gibbs sampler and its close relationship with the EM algorithm, we decided to use Gibbs’ sampler to implement the MCMC chain.

## Results

### Simulation results

**Fig 1:**
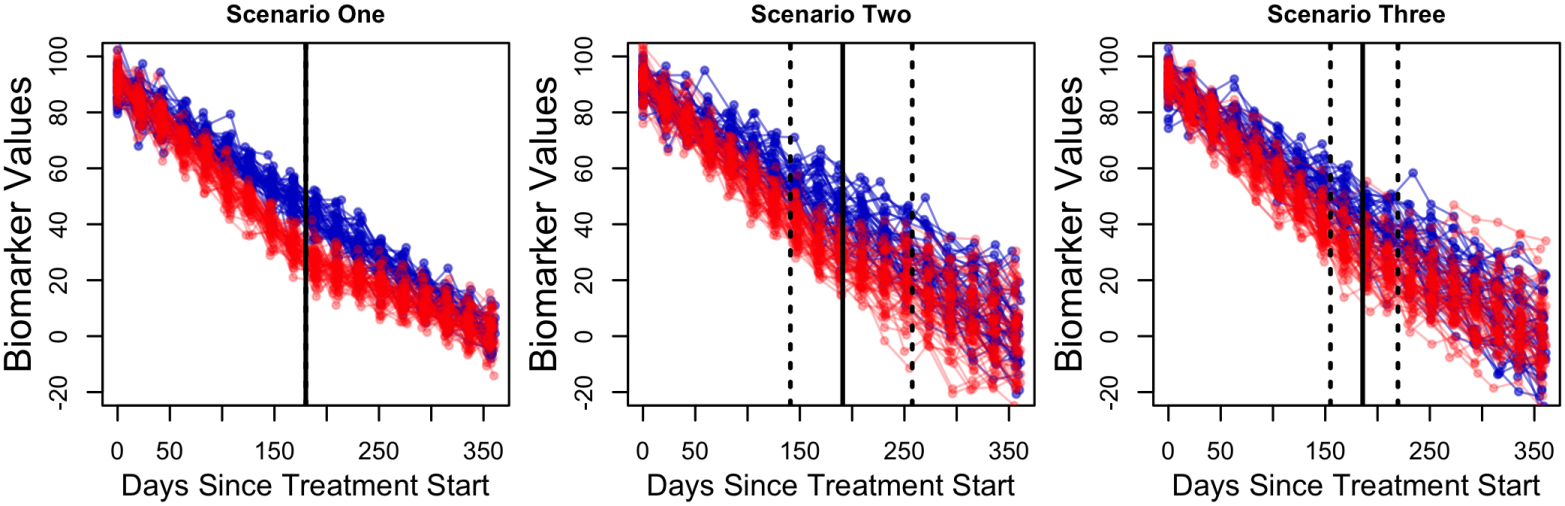
Longitudinal trajectories for the simulated patients in the three scenarios. Blue lines indicate mono-phasic patients’ trajectories, and red lines, bi-phasic patients’ trajectories. Vertical solid lines indicate the median time at which phasic transitions occur for the bi-phasic patients; vertical dashed lines indicate the 10^*th*^% and 90^*th*^% phasic transition time. All bi-phasic patients have the same phasic transition time in scenario one; hence, the dashed and solid lines coincide.

We designed three simulation studies to test the model’s abilities to categorize patients and to estimate associated parameters, Fig 1. All three scenarios have the same population-level regression parameters, as shown in Table 1; the differences between the three scenarios lie in the covariance matrices specifying between-patient variability. The first simulation scenario assumes that there is no between-patient variability; for the second scenario, there is between-patient variability in the intercepts and slopes in both mono- and bi-phasic patients but no correlation among these parameters, *i.e.* all non-diagonal entries in Σ_*s*_ and Σ_*b*_ are zero. The third scenario assumes a correlation of 0.5 between the first and second slopes among the bi-phasic patients. In each scenario, we simulated *N* = 100 patients. The probability of being a bi-phasic patient is *λ* = 0.60. According to a hypothetical clinical protocol, patient data are collected every 21 days with 1 at baseline and 17 at follow-up visits for a total follow-up duration of 357 days. In this simulation study, the actual visit time may deviate within ± 5 days from the scheduled time. The true individual regression parameters are drawn from multivariate normal distributions with respective population parameters and covariance matrices, (*S*, Σ_*S*_) or (*B*, Σ_*B*_), depending on patients’ phasicity. For each simulated bi-phasic patient *i*, the phasic transition time occurs at $t=\frac{b_{i0}-b_{i0}^{\prime }}{b_{i1}^{\prime }-b_{i1}}$.

**Table 1:** The true and the means of the estimated parameters in the three simulation scenarios.

	*S*_0_	*S*_1_	*B*_0_	*B*_1_	$B_{0}^{\prime }$	$B_{1}^{\prime }$	*σ*	*λ*
Truth	90	−0.25	91	−0.35	55	−0.15	5.0	0.6
Scenario One	90 (0.33)	−0.25 (0.001)	91 (0.19)	−0.35 (0.002)	57 (1.2)	−0.16 (0.004)	4.9 (0.066)	0.60 (<0.001)
Scenario Two	90 (0.43)	−0.257 (0.005)	90.6 (0.49)	−0.347 (0.006)	56.9 (1.14)	−0.150 (0.005)	5.0 (0.10)	0.50 (0.031)
Scenario Three	89 (0.41)	−0.250 (0.002)	90.8 (0.09)	−0.346 (0.005)	57.5 (0.20)	−0.157 (0.002)	5.1 (0.09)	0.53 (0.007)

The means of the EM estimated parameters over 1000 simulations are shown for each scenario. Standard deviations are shown in parentheses.

We first applied the EM algorithm to estimate the parameter values that maximize the marginal likelihood. The true and estimated parameters, excluding the covariance for the three scenarios, are shown in Table 1. In all three scenarios, the proposed model was able to provide parameter estimates that are close to the true parameter values, except for the bi-phasic proportion parameter *λ*, which is biased towards the mono-phasic model in scenarios 2 and 3. Estimating the covariance matrices for scenarios two and three is more challenging, as shown in Table 2. In particular, we found that the proposed model consistently over-estimates the variance term associated with the second intercept for the bi-phasic patients. Three possible causes for this over-estimation are 1) the bi-phasic design matrices must be estimated and misclassification of observations between the first and second phases may result in an enlarged variance term for the second intercept; 2) estimation of the second intercept requires projection back to time zero, and any uncertainty is magnified by this projection; and 3) the phasic transition time in our simulated data is distributed according the Gaussian ratio distribution, with heavy tails [35]; thus, bi-phasic patients with extreme transition times may not be classified correctly. In addition to parameter estimation, the proposed method performed well in classifying patients according to their phasicities, as shown in Table 3

**Table 2:** The true and the means of estimated covariance components in the three simulation scenarios.

Scenario 1					
	*b*_0_	*b*_1_	$b_{0}^{\prime }$	$b_{1}^{\prime }$	
	0 (1.0)	0 (−0.007)	0 (0.242)	0 (−0.001)	*b*_0_
		0 (<0.001)	0 (−0.007)	0 (0.000)	*b*_1_
*s*_0_	0 (0.6)		0 (14.8)	0 (−0.051)	$b_{0}^{\prime }$
*s*_1_	0 (−0.003)	0 (<0.001)		0 (<0.001)	$b_{1}^{\prime }$
	*s*_0_	*s*_1_			
Scenario 2					
	*b*_0_	*b*_1_	$b_{0}^{\prime }$	$b_{1}^{\prime }$	
	4 (6.1)	0 (−0.019)	0 (1.43)	0 (−0.005)	*b*_0_
		0.0009 (0.0006)	0 (−0.011)	0 (<0.001)	*b*_1_
*s*_0_	4 (4.5)		4 (14.8)	0 (−0.051)	$b_{0}^{\prime }$
*s*_1_	0 (0.004)	0.0009 (0.0011)		0.0009 (0.0009)	$b_{1}^{\prime }$
	*s*_0_	*s*_1_			
Scenario 3					
	*b*_0_	*b*_1_	$b_{0}^{\prime }$	$b_{1}^{\prime }$	
	4 (3.4)	0 (−0.006)	0 (0.768)	0 (0.009)	*b*_0_
		0.0009 (0.0005)	0 (−0.014)	0.0005 (0.0005)	*b*_1_
*s*_0_	4 (4.1)		4 (10.1)	0 (−0.036)	$b_{0}^{\prime }$
*s*_1_	0 (0.011)	0.009 (0.001)		0.0009 (0.0009)	$b_{1}^{\prime }$
	*s*_0_	*s*_1_			

True values are shown outside of the parentheses and estimated values are shown inside the parentheses. The lower triangular components of the mono-phasic covariance matrix and the upper triangular components of the bi-phasic covariance matrix are shown. The means of the estimated covariance components are calculated based on 1,000 simulation runs for each scenario.

**Table 3:** Classification accuracy for the three scenarios.

Representative examples		Scenario 1		Scenario 2		Scenario 3	
Estimated		Truth		Truth		Truth	
		Mono-phasic	Bi-phasic	Mono-phasic	Bi-phasic	Mono-phasic	Bi-phasic
	Mono-phasic	40	0	40	7	40	1
	Bi-phasic	0	60	0	53	0	59
	Sensitivity/Specificity	100%	100%	100%	88%	100%	98%
Averages of 1,000 simulations		100%	100%	99.99%	82.57%	99.99%	88.40%

A representative example for each scenario and the averages of 1,000 simulation runs are shown. A hard cut-off for calling a patient mono- or bi-phasic is used based on the expected probabilities of being bi-phasic. Patients with expected bi-phasic probabilities exceeding 0.5 are classified to be bi-phasic, otherwise mono-phasic.

**Fig 2:**
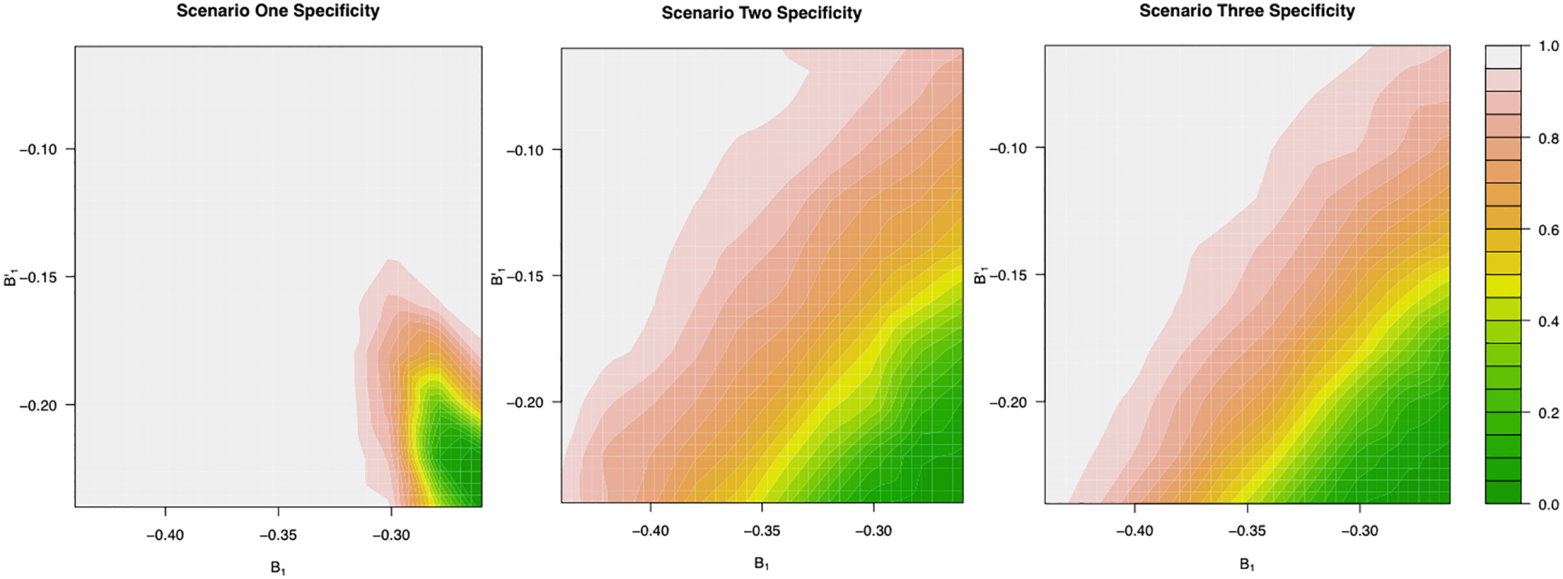
Specificity as a function of true bi-phasic slopes. True mono-phasic slope is kept at −0.25; bi-phasic first slopes vary between −0.45 and −0.26; bi-phasic second slopes vary between −0.24 and −0.05. Population-level mono-phasic slope and bi-phasic first intercepts are 90 and 91 respectively; the second slopes for bi-phasic patients are selected such that the population-level phase transition occurs at 178 days, which is in the middle of 357-day trial period. Each graph is generated based on the averages of 10 simulations. Sensitivity is omitted since it is at 100% for all given scenarios; please refer to Table 3.

**Fig 3:**
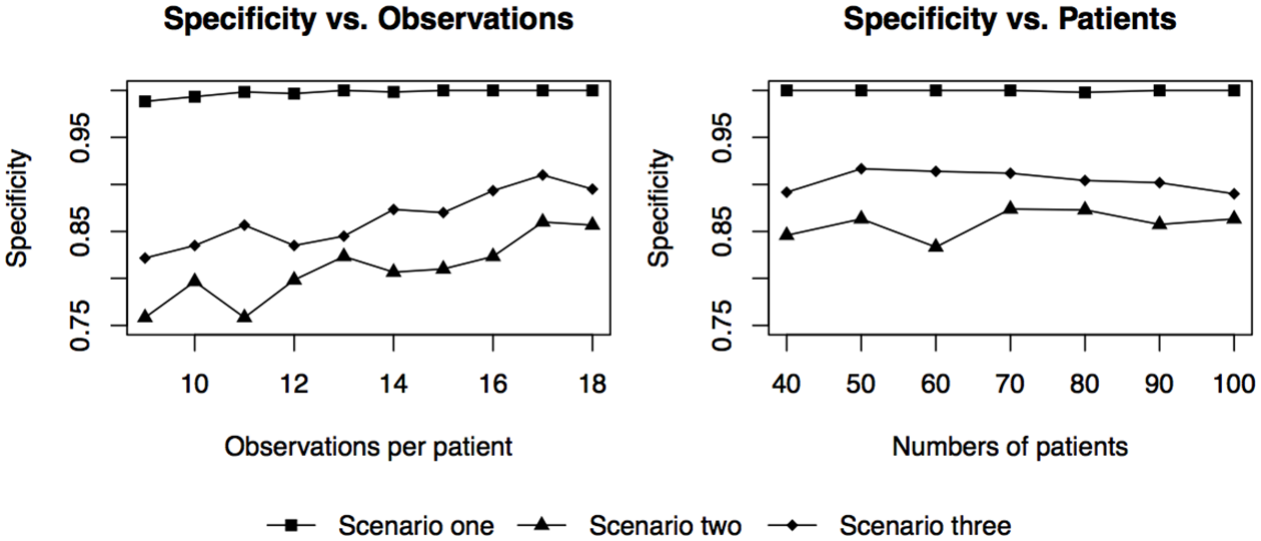
Specificity as a function of the numbers of observations per patient and the total numbers of patients per simulated data set. Parameter values are identical to those used in the three scenarios. The numbers of observations per patient vary in the figure on the left; these observations are evenly distributed between day 0 and day 357. The total numbers of patients, *N*, vary in the figure on the right; the number of observations per patient is kept at 18.

In addition to the three scenarios outlined above, we also performed sensitivity analyses to test the effects of variability in population-level intercepts and slopes, $S_{0},S_{1},B_{0},B_{1},B_{0}^{\prime },$ and $B_{1}^{\prime }$, on the classification accuracy, Fig 2. For each of the three scenarios, we tested a grid of values for the bi-phasic first slope, *B*_1_ (−0.45, …, −0.26), and the second slope, $B_{1}^{\prime }$ (−0.24, …, −0.05), centering around the mono-phasic slope *S*_1_ = −0.25. For this sensitivity analysis, the second intercept for the bi-phasic patients was kept at values such that the population-level phasic transition times occurred in the middle of the time span of the trial (178 days). All other parameters, *S*_0_, *S*_1_, *B*_0_, *σ* and *λ*, were kept at the values used in the previous three scenarios. The covariance matrices, if applicable, were also kept at the values used in the three scenarios. As expected, as the bi-phasic first and second slopes approached the value of the mono-phasic slope, the specificity diminished in all three scenarios such that more bi-phasic patients were misclassified as mono-phasic patients. Due to the strong penalty induced by the BIC correction in deciding on patients’ phasicities, the proposed model is biased toward the mono-phasic model. Sensitivity is close to 100% in all three scenarios; hence, the contour plots for sensitivity are not shown. In addition, we also investigated the effects of the numbers of observations per patient and the effects of the numbers of patients on the method’s ability to distinguish between mono- and bi-phasic patterns. As expected, as the number of observations per patient decreases, specificity decreases. Interestingly, the model is not very sensitive towards the total number of patients as shown in Fig 3.

We also compared our model and its estimates with a standard mixture model package, Flexmix, a publicly available package in R [36, 37]. For each simulation, we ran the Flexmix package with and without providing the true design matrix, and true mixture identity as the initial values. The true design matrix groups data points from the same phase together for each subject; the true mixture identity provides the initial clustering of data points from the same phase across different patients together. The means of the parameters from 1,000 simulations are shown in Table 4. Because the Flexmix package is not designed to estimate the change point, the primary comparison of interest is to compare Flexmix’s ability to identify and estimate parameters associated with the three components of the mixture: single-phasic, bi-phasic first phase, and bi-phasic second phase. For scenario 1, in which is no between-subject variability, without providing both true design matrix and mixture identity, the mixture model was not able to estimate the parameters accurately, as compared to our model Table 1. Providing the true design matrix and mixture identity greatly improves the mixture model’s ability to estimate the parameters; however, this improvement is only limited to the case in which there is no between-subject variability. Once between-subject variability is introduced in scenarios 2 and 3, the mixture model was not able to estimate these parameters correctly.

**Table 4:** Comparison of estimates from a mixture model with and without a true design matrix and true cluster identity using the Flexmix package.

			True Parameter Values							
Between-Subject Variability	True Design Matrix Provided	True Cluster Identity Provided	*S*_0_	*S*_1_	*B*_0_	*B*_1_	*B*_1_	*B*_2_	*σ*	*λ*
			90	−0.25	91	−0.35	55	−0.15	5.0	0.60
Scenario 1	No	No	86	−0.26	86	−0.31	89	−0.25	5.5	0.61
	No	Yes	85	−0.26	88	−0.33	88	−0.25	5.3	0.59
	Yes	No	90	−0.25	91	−0.35	57	−0.25	5.1	0.61
	Yes	Yes	90	−0.25	91	−0.35	55	−0.15	5.0	0.60
Scenario 2	No	No	89	−0.26	86	−0.30	90	−0.22	6.2	0.54
	No	Yes	89	−0.26	86	−0.30	90	−0.22	6.2	0.55
	Yes	No	87	−0.25	87	−0.31	87	−0.22	6.8	0.58
	Yes	Yes	89	−0.24	88	−0.30	65	−0.16	6.7	0.64
Scenario 3	No	No	88	−0.26	86	−0.30	89	−0.22	6.3	0.54
	No	Yes	88	−0.26	86	−0.31	90	−0.22	6.3	0.54
	Yes	No	87	−0.26	87	−0.31	88	−0.21	6.8	0.60
	Yes	Yes	89	−0.24	88	−0.31	64	−0.16	6.8	0.61

The means of the estimates from 1,000 simulation runs are shown.

In addition to obtaining the maximum likelihood parameter estimates, Gibbs’ sampling was implemented to obtain the posterior densities for the estimated parameters for the three scenarios. The key parameter of interest in this simulation study is the coverage probability for the proposed model. We simulated 1,000 independent data sets using identical parameter values for each scenario. The EM algorithm was first applied to maximize the likelihood; using the maximum likelihood parameter estimates as starting values, a Markov Chain Monte Carlo simulation was performed for each data set, with the number of iterations per simulation equal to 30,000. With samples generated from the posterior distributions, we constructed a 95% simultaneous rectangular credible region for each simulated data set, using the method outlined by Held [38, 39]. The coverage probability is calculated as the probabilities of the simultaneous credible regions covering all 8 parameters for scenario one and covering all 21 parameters for scenarios two and three, out of the 1,000 simulated data sets. The coverage probabilities are 80.1%, 71.5% and 65.9% for the three scenarios, respectively. The convergence of the Gibbs’ sampler is shown in S1 File.

We then performed detailed analyses to determine the parameter with the worst coverage probability in each scenario. For scenario one, the second intercept for the bi-phasic patients, $B_{0}^{\prime }$, had the worst coverage probability of only 80.1%. Further analyses of scenarios two and three revealed that the variance component for the bi-phasic second intercept had the lowest coverage rate among all parameters; the 95% simultaneous credible regions for all 21 parameters were able to cover the covariance term for the second intercept only at rates of 82.5% and 80.1% for scenarios two and three, respectively. In this case, the covariance components obtained from the Gibbs’ sampler were consistently higher than the true covariance used in the simulated data. The same reasons used to explain the enlarged covariance structure for the EM results can be applied here.

Another parameter of particular interest is the correlation between the first and second slopes in scenario three. Focusing only on this parameter, our model was able to detect a correlation in 62.1% of the simulation runs; detection refers to 0 being excluded from the 95% credible region. Overall, the actual coverage probabilities from the proposed model are lower than the nominal probabilities. Model complexity appears to contribute to this poor coverage, as previous research has shown that even in the simple binomial case, the coverage probability rarely agrees with the nominal probability [40]. In addition, the parameter values, particularly the slopes, used in our simulation have considerable overlaps, which renders identifying patients’ phasicities difficult, and hence lowers the coverage probabilities.

### Application results

**Fig 4:**
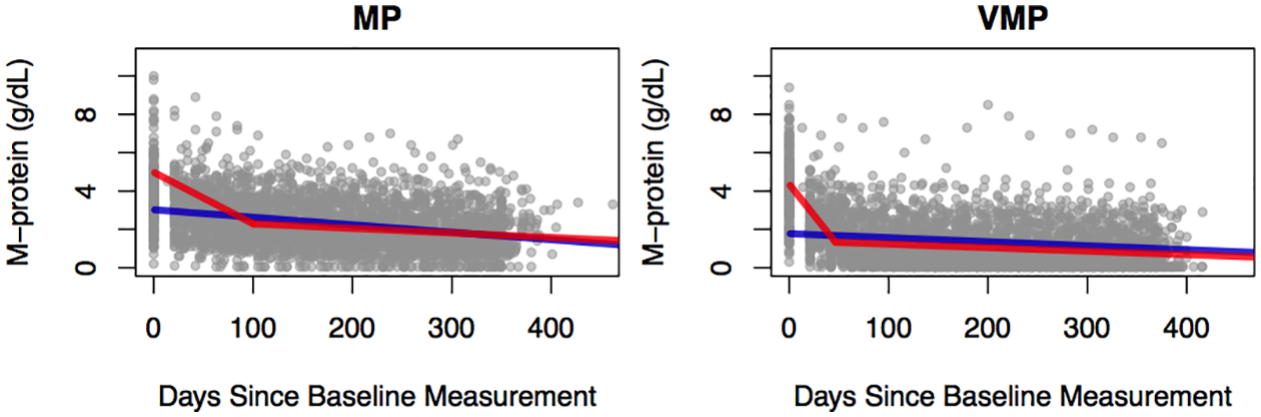
Longitudinal trajectories for patients in the VISTA trial separated by treatment cohorts. The mono-phasic (blue) and bi-phasic (red) lines indicate the population-mean trajectories based on the maximum likelihood estimates from the EM algorithm.

To further demonstrate the utility of the proposed methods, we applied it to the M-protein data from the Velcade as Initial Standard Therapy in Multiple Myeloma: Assessment with Melphalan and Prednisone (VISTA) trial [41]. Briefly, the VISTA trial is a randomized, open-label phase III study, consisting of 682 patients with newly diagnosed, previously untreated, symptomatic, measurable multiple myeloma. In this study, patients were randomized to treatment with either melphalan and prednisone with (VMP cohort) or without (MP cohort) bortezomib (Velcade, Johnson & Johnson Pharmaceutical R&D and Millennium). Measurable disease was defined as the presence of quantifiable M-protein in serum or urine, or measurable soft-tissue or organ plasmacytomas. The longitudinal M-protein data from patients in the VISTA trial are shown in Fig 4.

The parameter estimates from our model revealed several interesting features associated with the M-protein dynamics, Table 5. First, the differences between the first and second slopes for the bi-phasic patients in both cohorts are striking. For the bi-phasic patients, the gradient of the first slope was lower than the second slopes in both cohorts, judging by the posterior credible regions. Second, more patients in the VMP cohort displayed bi-phasic trajectories than in the MP cohort. Third, the gradient of the bi-phasic first slope in the VMP cohort is lower than that of the bi-phasic first slope in the MP cohort. Fourth, the bi-phasic first intercepts are similar in both cohorts. Fifth, the long-term declines for the bi-phasic patients in both cohorts are similar. Sixth, in both cohorts, the intercepts for the mono-phasic patients tend to be substantially smaller than the first intercepts of the bi-phasic patients. Lastly, in the MP cohort, despite the large differences in the rates of initial declines, the long-term declines are very similar between the mono-phasic and the bi-phasic patients, as shown by the similarity in the estimates for *S*_1_ and $B_{1}^{\prime }$. Those observed differences in the M-protein dynamics between cohorts suggest that the tumor dynamics of multiple myeloma are highly complex.

**Table 5:** 95% simultaneous credible regions for the MP and VMP cohorts in the VISTA trial.

Cohorts	*S*_0_	*S*_1_	*B*_0_	*B*_1_	$B_{0}^{\prime }$	$B_{1}^{\prime }$	*σ*	*λ*
MP	(2.34, 3.06)	(−0.004, −0.002)	(4.35, 5.46)	(−0.036, −0.016)	(2.23, 3.20)	(−0.005, −0.001)	(0.25, 0.29)	(0.359, 0.592)
VMP	(1.06, 2.67)	(−0.003, −0.000)	(4.00, 4.70)	(−0.077, −0.053)	(1.12, 1.69)	(−0.003, −0.001)	(0.22, 0.34)	(0.794, 0.938)

## Discussion

We have proposed a piecewise linear mixture random-effects model to investigate the extent of heterogeneity and time-varying functional relationships in longitudinal biomarker data. The combination of heterogeneity and a time-varying functional relationship is where the innovation in the proposed model lies. Our model assumes a simple yet robust piecewise linear functional form. The major advantage of this piecewise linear functional form over other more complex nonlinear functions is that the likelihood can be maximized analytically, using empirical Bayes estimators and standard expectation-maximization algorithms. No prior knowledge of the functional relationship other than the piecewise assumption is required; thus this method is particularly useful for initial exploratory analyses. In addition, the ease of interpretation of the parameter estimates is another advantage of the proposed model. Lastly, in the extreme case in which all patients are mono-phasic, the proposed model completely reduces to linear mixed-effects model.

One minor drawback of our approach is that for the bi-phasic patients, the proposed model produces a point of discontinuity between *k*_*i*_ and *k*_*i* + 1_ observations Eq (1). Nonlinear models, such as the broken-stick model, Bacon Watts model, and the polynomial model suggested by Matthews *et al.* offer potential solutions to this problem [42]; however, analytical solutions do not exist for those nonlinear functions. Another minor problem is that there is a small bias for the parameter denoting the proportion of bi-phasic patients, *λ*, in the EM algorithm and Gibbs’ sampler. Closer investigation reveals that this bias is not due to our proposed model; rather, it is an artifact of the data generation process for the simulation studies (see S1 File for detail). The simulated data for the bi-phasic patients are generated using a multivariate normal distribution. An unwanted implication of this generation mechanism is that the phasic transition time follows a Gaussian Ratio distribution, with heavy tails, such that for some bi-phasic patients the actual transition time may exceed the window of observation. This problem is particularly confounding in the case in which the first and second slopes are close in value to the slope of the true single-phasic slope parameter. As a result, such bi-phasic patients are indistinguishable from single-phasic patients. Thus, this small bias indicates that our proposed method was able to classify these bi-phasic patients “correctly” as single-phasic, based on the observed data. Fig A in S1 File shows a few such examples. The four figures in the bottom left corner of Fig A in S1 File are from true bi-phasic patients; however given the late phasic transitions and steep second slopes, our algorithm is likely to classify them as mono-phasic. This “misclassification” is due to the similarity of those patients’ trajectories to those mono-phasic patients rather than a systematic mistake in our algorithm. Lastly, the MCMC algorithm requires a large sample size to be implemented due to its model complexity—21 parameters in total. We recommend a sample size of at least 100 patients and with sufficient numbers of mono- and bi-phasic patients, greater than 40 each, to ensure that the number of patients is greater than the number of parameters.

When applying this algorithm to a data set from the VISTA trial, although from our analysis we have not found a significant correlation between phasicities and patients outcomes, the distinct mono- and bi-phasic trajectories may have significant medical implications warranting further investigation and validation. Those findings on the distinct treatment responses for patients randomized to the same treatment arm may help generate new hypotheses for improving patient prognosis and disease management.

From the prospective of clinical trial design, one interesting question is how to design a trial to maximize phasicity detection, if phasicity is important for patient management. Our linear framework may offer a simple approach to address these issues. In addition, our model can be extended beyond between-patient variability to include additional layers inside the hierarchy. For instance, patients with metastatic solid tumors or multiple tumors at different sites may demonstrate a large degree of similarity in terms of individual tumor trajectories, yet also exhibit kinetic differences depending on an individual tumor’s microenvironment. Modeling treatment responses in such scenarios would then require the incorporation of between-patient variability and within-patient/between-tumor variability into the model. Furthermore, our model can be extended to include multi-category and multi-phasic changes. The computational tractability problem associated with multi-phasic changes can be addressed from a practical point of view, such as restricting the number of minimum observations in each phase to be greater than 5 data points. These additional extensions can further enrich our proposed model.

## Supporting information

S1 File EM starting value search algorithm, additional EM examples, and assessment of Gibbs’ sampler convergence. (PDF)
