# Supplementary material for: Estimating mono- and bi-phasic regression parameters using a mixture piecewise linear Bayesian hierarchical model
Source: PLoS One. 2017 Jul 19;12(7):e0180756. doi: 10.1371/journal.pone.0180756 (PMC5516991; doi:10.1371/journal.pone.0180756)
Supplement: S1 File — (PDF) [file pone.0180756.s001.pdf]

S1 File: EM starting value search algorithm, additional EM examples, and assessment of Gibbs' sampler convergence.

1 Procedure for determining initial values:

Figure A shows some representative examples of mono- and bi- phasic patients used in our simulation studies. Note the similarity between mono- and bi- phasic trajectories among those patients. To search for the starting values for the EM algorithm, S, B, Σ_S, Σ_B, λ and σ^2, an *ad hoc* grid search procedure was used. For the mono-phasic patients, the starting values were estimated by fitting a simple linear regression for each patient, regardless of the true phasicity. The maximum and minimum values of intercepts and slopes define the range of the starting values for the EM algorithm. The starting values for the mono-phasic between-patient covariance matrix was obtained by calculating the covariance between simple linear regression parameters. For the bi-phasic starting values, the 0th to 30th percentiles of observations and the 70th to 100th percentiles of observations, ranked according to the time of measurement, were used to establish the search space for the parameters of the first and second phases. The search space for the first and second slopes is between the estimates obtained from the first 30 percentiles of the observations and the last 30 percentiles of the observations. Likewise, the covariance for these parameters was used as the starting values for the between-patient covariance matrix. The standard deviation for the noise parameter, σ, was obtained from fitting a simple linear regression to all data points across all patients. The proportion parameter, λ, was drawn from a uniform distribution, (0.2, 0.8). We truncated values from below 0.2 and above 0.8 to prevent the algorithm from classifying all patients as either mono- or bi-phasic based on the starting value. The cases for which λ is below 0.2 are not considered in our samples, since in those cases, the number of patients displaying bi-phasic patterns is too small to estimate all parameters for the bi-phasic patients. Similarly, the cases for which λ

is above 0.8 are not considered, since there may not be enough mono-phasic patients to estimate the mono-phasic parameters. However, as the number of patients increases, the search space range can be modified to be closer to 0 and 1. In addition, a few *ad hoc* rules were implemented to filter out unreasonable starting values; for example, bi-phasic values leading to a transition point before the first data point or after the last data point were excluded. In addition, in our simulations, we only considered the scenarios in which the mono-phasic slope is sandwiched between the bi-phasic first and second slopes. The parameter values that yield the highest marginal likelihood is used as the starting value for the MCMC chain. Figure B shows the convergence of the EM algorithm to a local maximum.

2 Additional Examples of the EM Algorithm:

We provide additional EM algorithm examples to demonstrate that our model's ability to distinguish between single- and bi-phasic patients and to estimate their parameters is not due to the difference in these patients' intercepts at time zero. These examples, shown in Table A, are similar to those shown in Table 1 of the main text, except for the intercepts for the bi-phasic patients, which were changed from 91 to 90.

Table A: The true and the means of the estimated parameters in the three simulation scenarios. The means of the estimated parameters are calculated based on 100 simulation runs for each scenario.

	S_0	S_1	B_0	B_1	B_0'	B_1'	σ	λ
Truth	90	-0.25	90	-0.35	55	-0.15	5	0.6
Scenario 1	90.0	-0.250	89.9	-0.347	56.1	-0.154	4.89	0.60
Scenario 2	89.8	-0.257	90.4	-0.345	56.9	-0.150	4.99	0.51
Scenario 3	89.7	-0.254	90.7	-0.345	56.9	-0.154	4.99	0.54

3 Convergence of Gibbs' Sampler:

The convergence of the Gibbs sampler was assessed using graphical methods as well as Gelman and Rubin diagnostics [1]. The trace plots and autocorrelation plots for an MCMC chain generated under simulation scenario 2 are shown in Figure C and Figure D. For the Gelman Rubin diagnostics, 5 independent MCMC chains with different starting values centered at the posterior mode were generated. The plots for the Gelman Rubin diagnostic are shown in Figure E. These figures show that the MCMC chains converge and are well mixed.

References

[1] Gelman A, Rubin DB. Inference from iterative simulation using multiple sequences. Statistical science. 1992; p. 457–472.

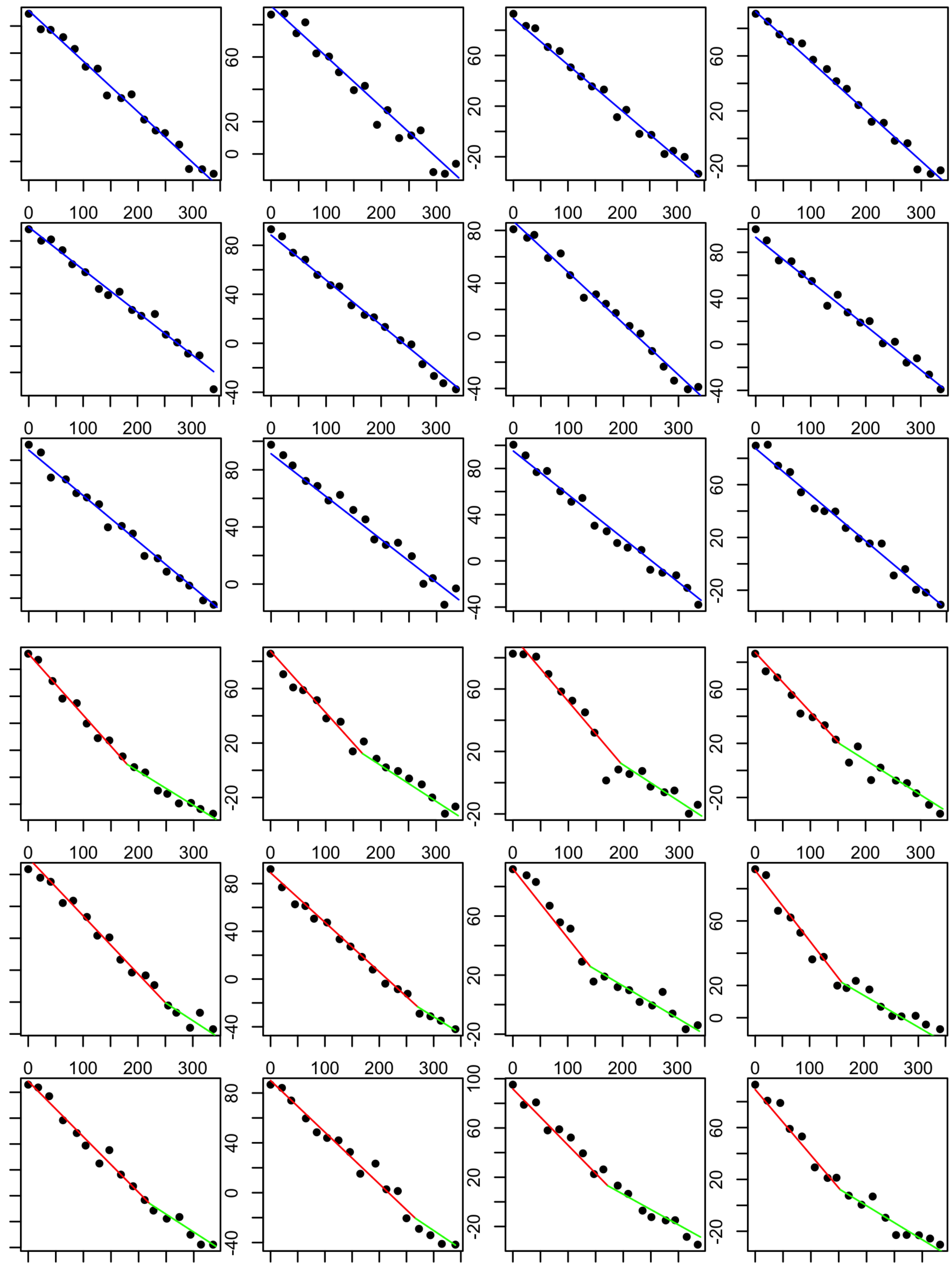

Figure A: Examples of simulated subjects. The first 12 subjects are mono-phasic and the last 12 subjects are bi-phasic. The true regression lines are shown.

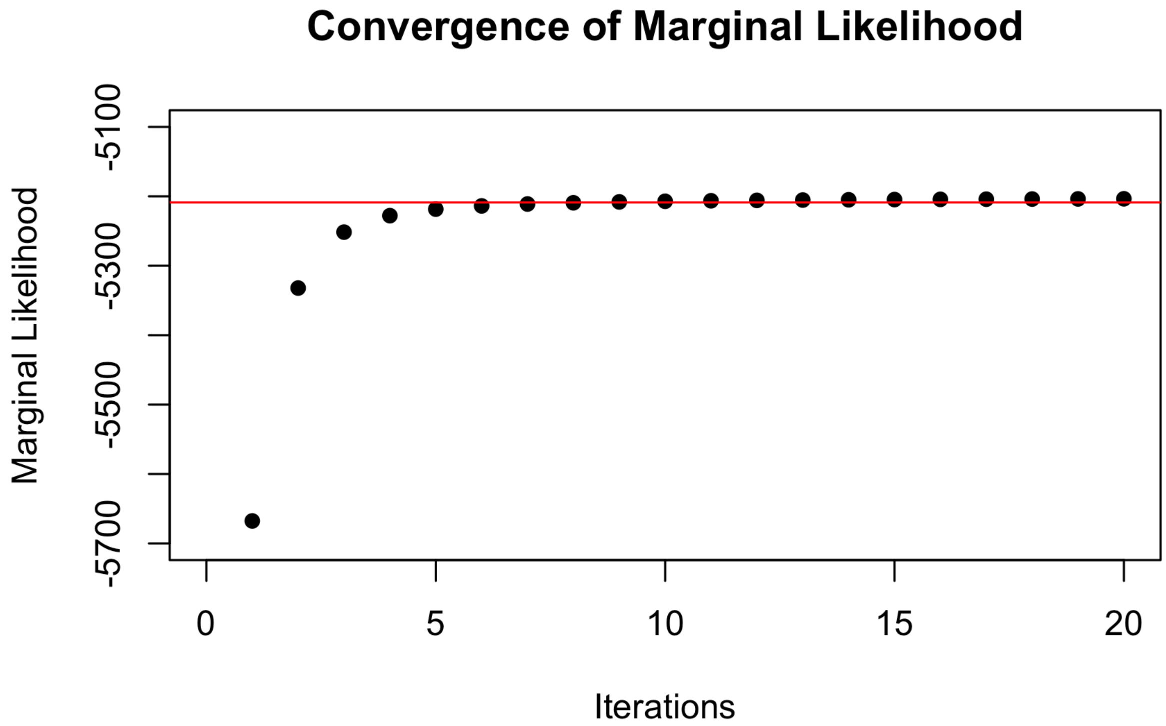

Figure B: Convergence of the marginal likelihood using starting values that produce the highest marginal likelihood among all combination of starting values. The red line denotes the true likelihood value.

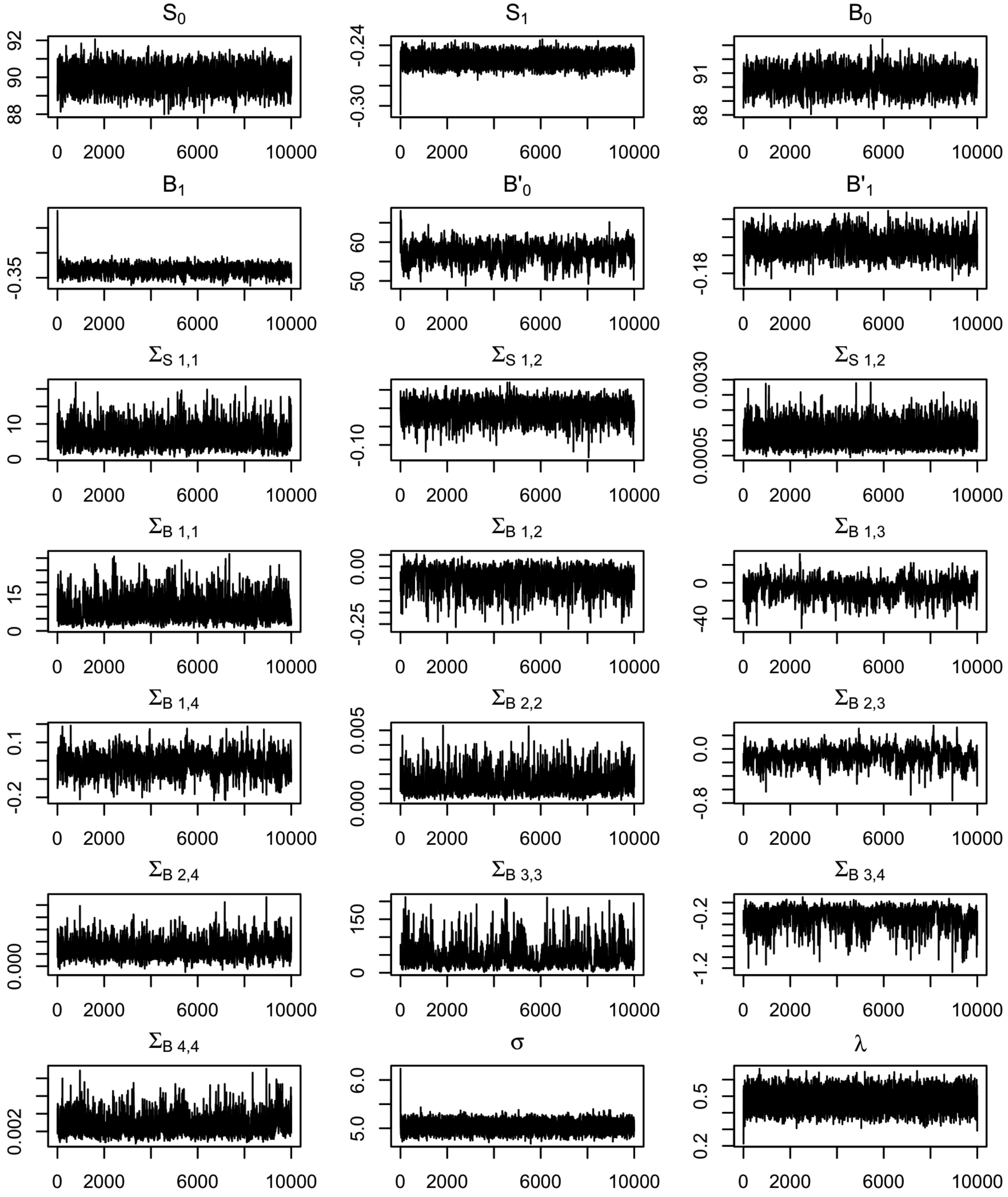

Figure C: Trace plots

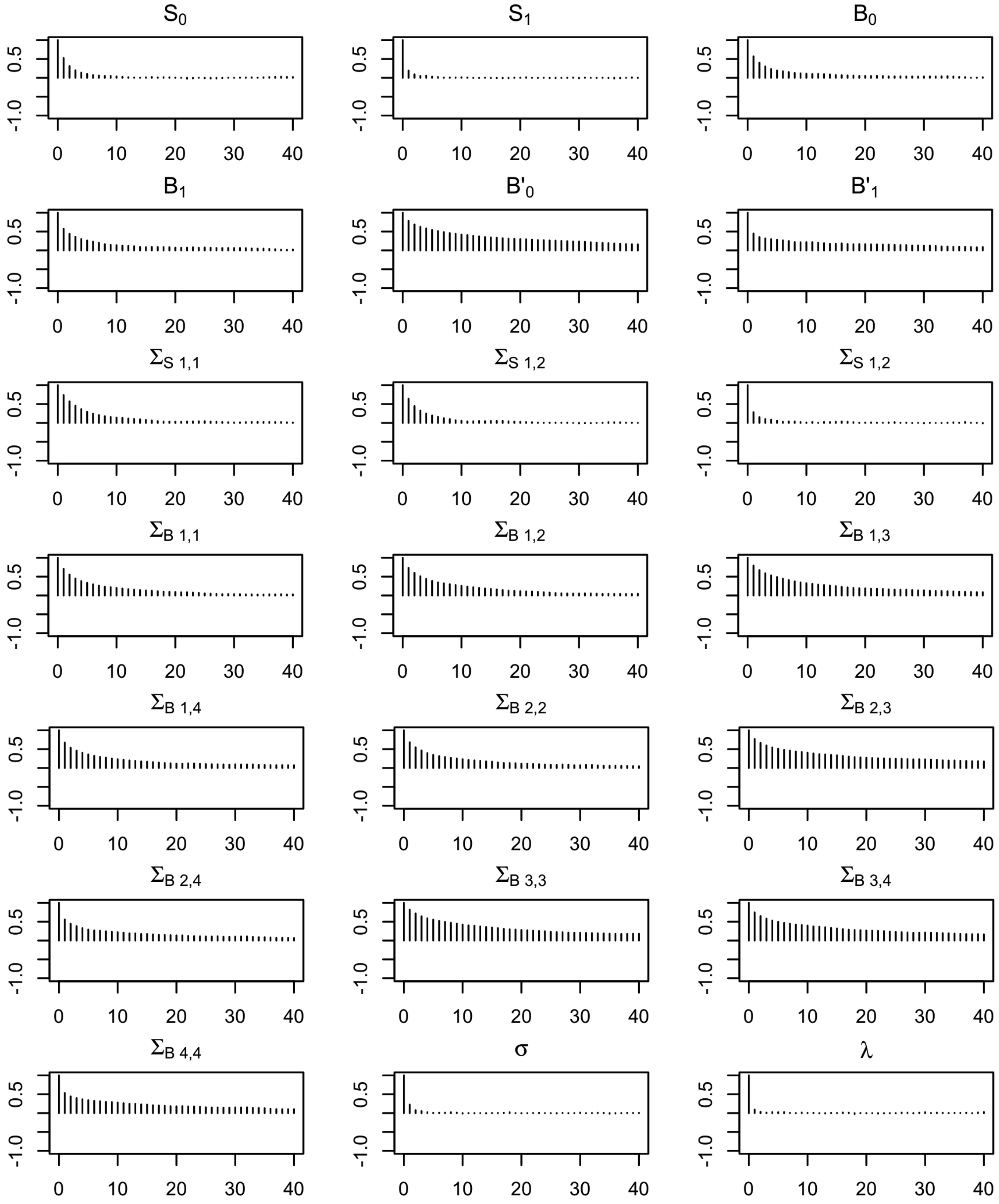

Figure D: Autocorrelation plots

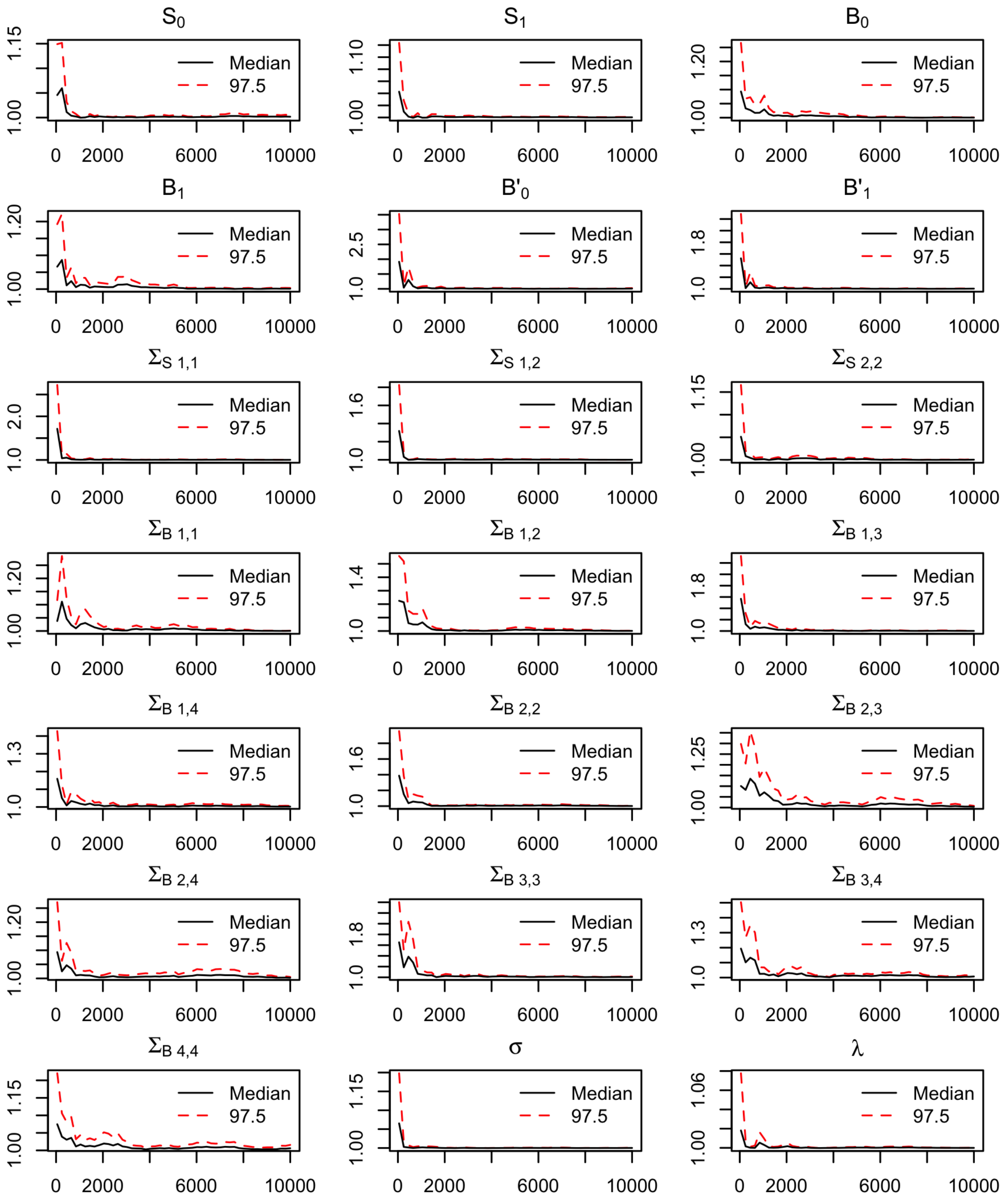

Figure E: Gelman and Rubin diagnostic plots
